# Appraisal of the knowledge, attitude, perception and practices among northern Nigerians in the wake of the COVID-19 outbreak

**DOI:** 10.2144/fsoa-2021-0058

**Published:** 2021-12-01

**Authors:** Nafi'u Lawal, Muhammad Bashir Bello, Yusuf Yakubu, Aliyu Musawa Ibrahim, Samira Anka Rabiu

**Affiliations:** ^1^Department of Veterinary Microbiology, Faculty of Veterinary Medicine, Usmanu Danfodiyo University, Sokoto, Nigeria; ^2^Central Veterinary Research Laboratory, Faculty of Veterinary Medicine, Usmanu Danfodiyo University, Sokoto, Nigeria; ^3^The Fleming Fund Antimicrobial Susceptibility Testing Laboratory, Veterinary Teaching Hospital, Faculty of Veterinary Medicine, Usmanu Danfodiyo University, Sokoto, Nigeria; ^4^Department of Veterinary Public Health & Preventive Medicine, Faculty of Veterinary Medicine, Sokoto, Nigeria; ^5^Center for Advanced Medical Research & Training, College of Health Sciences, Usmanu Danfodiyo University, Sokoto, Nigeria; ^6^Department of Science & Vocational Education, Faculty of Education & Extension Services, Usmanu Danfodiyo University, Sokoto, Nigeria

**Keywords:** attitude, COVID-19, face masks, hygienic practices, knowledge, perception, practice, SARS-Cov2

## Abstract

**Aim:** The aim of this study was to measure the knowledge, attitude, perception and practices of northern Nigerians toward the COVID-19 pandemic. **Materials & methods:** This was a questionnaire-based cross-sectional study and the data were analyzed using descriptive and inferential statistics. **Results & discussions:** There were 713 participants, of which 54.0, 57.4, 67.6, 36.2 and 28.9% were between 18 and 30 years of age, married, males, having bachelor's degree and civil servants, respectively. High level of knowledge, attitude, perception and practice was found. Pearson correlation analysis found strong positive (r = 0.622; p < 0.001) relationships between knowledge, attitude, perception (r = 0.454; p < 0.001) and at last, practice (r = 0.282; p < 0.001). **Conclusion:** Young, male and married northern Nigerians of high socio-economic status had better knowledge, attitudes, perceptions and practices toward COVID-19.

## Lay abstract

The COVID-19 pandemic outbreak is still affecting the world with infections still soaring. The prevention and control strategies still remain: personal hygienic practices, vaccination, avoidance of overcrowding and wearing of face masks outdoors. Full compliance and adherence to these control measures is determined by the level of knowledge, attitude, perception and practices on possession of the disease, which is what this study sets to measure. We found that young, male, married, educated and employed people have higher level of knowledge, positive attitude, good perception and practices toward COVID-19.

## Background

An epidemic pneumonia of unknown etiology emerged in the city of Wuhan, China in December of 2019. A novel coronavirus named SARS-CoV-2 was identified as the causative agent of COVID-19, after its genome had been fully sequenced and analyzed [1–3] as the new member of the Coronaviridae, a family of viruses with positive-sense, single-stranded RNA genome [4]. Since then the disease has been spreading globally [2,3] and on 23 February 2020, the Nigeria Center for Disease Control (NCDC) announced the confirmation of the first imported case of COVID-19, thereby becoming the third African country after Algeria and Egypt to be affected by the outbreak. As at 3 April 2020, the number of confirmed cases in the country had risen to 190, with two deaths recorded. Among the victims of the disease were some prominent and highly placed Nigerian politicians and/or their family members, causing panic among the citizenry that lead to the suspension of some administrative and economic activities. The disease is highly contagious with high morbidity. Infected patients present clinical signs such as fever, dry cough, dyspnea, myalgia and fatigue [1,5]. There are three clinical presentations of the disease; the mild form which constitutes about 80.9% of the cases presenting with mild, limited or no symptoms at all [6]; the severe and the critical forms which together constitute 19.1% of the clinical cases. Although the first cases were detected in December 2019 the disease quickly spread so much so that by March 2020 it was detected in nearly all the continents of the world [5,7]. The overall laboratory confirmed cases as of 28 March 2020 were 512,701 with 23,495 confirmed mortalities from 202 countries, areas or territories. In an effort to curtail the spread of this infection, 19 northern Nigerian governors declared that all state schools at all levels within their states will be closed by 23 March 2020 and interstate travel be reduced to the minimum to conform with the global practice in the control of COVID-19 [8]. The federal government followed suit by closing down all federal higher institutions of learning and all international airports. Nigerian citizens were also strongly advised through media campaigns to stay at home, avoid large gatherings and maintain personal hygiene, especially frequent hand washing with soap or hand sanitizers. The COVID-19 outbreak if unchecked would stretch the health capacity of even developed economies like USA and China, let alone resource-limited countries like Nigeria. To succeed in the fight against the spread of the disease, the control measures set by government must be strictly adhered to, and this is determined by their knowledge, attitudes, perception and practices (KAPP) toward the disease, COVID-19. In this comparative study, the KAPP of the northern Nigerians toward COVID-19 was investigated and compared based on their demographic features to determine their understanding on the critical role they will play in the management and control of this pandemic and to aid in determining the efficacy of the massive COVID-19 awareness campaigns carried out by the government of Nigeria.

## Materials & methods

### Study participants

The study is a cross-sectional design conducted for 1 week, from 28 March 2020 to 4 April 2020, following the method described by [9,10] with little modification. Because of the curfew imposed by some northern states in the country, it is not feasible to administer hard copies of the questionnaire instrument in such states. Online platforms such as emails, WhatsApp and Telegram through the authors' personal network and connections was used to administer the questionnaire as previously described [9]. In such states where movement restrictions were not strictly imposed, hard copies of the questionnaire were distributed to the respondents. No sample size was calculated for this study because of the limitations of lockdown policy, but we tried to reach out to as many respondents as possible. According to Krejcie and Morgan's table for sample-size calculation, 400 samples are adequate for any given population that reach 1 million and above. The sampling technique used was purposive. A section with brief introduction, objectives of the study, informed consent and declaration of confidentiality and anonymity were attached to the questionnaire for the participants to see. The inclusion and exclusion criteria used to recruit the participants were as follows: any person male or female of 18 years and above, residing in Northern Nigeria and who is willing to participate through informed consent by answering ‘yes’ for the ‘yes or no’ questions was included in the survey and any person below 18 years of age, not residing in Northern Nigeria or not willing to participate in the study was not included.

### Questionnaire instrument

The questionnaire was divided into sections 1 and 2 as previously described [9]. Section 1 contained the respondent's demographics such as: gender, age, marital status, place of residence, level of education and occupation. Section 2 contained the KAPP questions.

According to the WHO's information and guidelines on the clinical presentation, management and prevention measures of COVID-19 and the NCDC guidelines on COVID-19 case definition, management and prevention and control guidelines, a knowledge questionnaire with 15 questions (K1–K15) was developed by the author and was answered as true (T) or false (F). K1–K3 was about the COVID-19 etiology, K4–K7 was about the clinical presentations, K8–K11 was about the transmission, K12–K15 was about prevention and control measures. One point is assigned to any correct answer given and 0 points were assigned to any incorrect or ‘I don't know' answers. The knowledge score obtained for each respondent was recorded from 0 to 15, and the higher the score, the better the knowledge of COVID-19.

The attitude (A) of the respondents toward COVID-19 disease was measured by five questions (A1–A5) on true or false basis. Each response was scored on a scale of 0 and 1 with the score of 0 for incorrect or 'I don't know’ answers and score of 1 for correct answers, respectivel. The attitude score for each respondent was recorded from 0 to 5. The perception (P) of the respondents on COVID-19 was measured using five questions (P1–P5) on the basis of true or false or 'I don't know' about the origin of COVID-19 which was scored 1 for correct answer and 0 for incorrect and 'I don't know' answers, respectively. The perception score for each respondent was recorded from 0 to 5. The practices (Pr) of the respondents were measured using five questions (Pr1–Pr5) based on their adherence to the control measures to stop the spread of COVID-19 in the population. The practice score for each respondent was recorded from 0 to 5 (Table 1) and scored on the same scale as for attitude and perception.

**Table 1.** Questionnaire instrument used to measure knowledge, attitude, perception and practice of the respondents.

S/number	Questions	T	F	IDK
Knowledge				
K1	The cause of COVID-19 is SARS-Cov-2	(51.8%)		
K2	It is closely related with bat coronavirus	(57.9%)		
K3	It is an RNA virus, so it can easily mutate its genome	(58.1%)		
K4	Coronaviruses can easily mutate in their genome	(88.2%)		
K5	High fever, dry cough, myalgia and respiratory distress are the main clinical signs of COVID-19	(89.1%)		
K6	Currently there is no effective cure or vaccine available for COVID-19, but early treatment symptomatically with supportive therapy can help most patients recover	(79.2%)		
K7	About 80% of COVID-19 patients will present with the mild form	(73.5%)		
K8	Elderly, immunocompromised people or those with comorbidities such as diabetes have higher risk of coming down with the severe and critical form of COVID-19	(74.5%)		
K9	Contact with wild animals may increase the chances of contracting COVID-19	(55.1%)		
K10	COVID-19 virus can stay suspended in air for 3 h	(65.6%)		
K11	COVID-19 can be transmitted through aerosol when infected person cough or sneezes without covering the mouth	(90.2%)		
K12	Hand shake with COVID-19 patient's contaminated hand can cause transmission of infection	(95.1%)		
K13	The use of surgical mask can reduce the transmission of COVID-19	(84.7%)		
K14	Patients with COVID-19 infection can infect at least 2.6 persons per transmission cycle if protective measures are not employed	(74.6%)		
K15	People should avoid large gathering and any person who have unprotected contact with COVID-19 patient should be immediately isolated and observed for 14 days	(95.0%)		
Attitudes				
A1	Social distancing can help stop the COVID-19 outbreak	(87.2%)		
A2	Frequent hand washing with sanitizers or soap will break COVID-19 transmission	(92.6%)		
A3	Most of our hospitals are not adequately equipped to handle COVID-19 patients with the severe form of the disease	(87.2%)		
A4	People with history of recent oversea travel that did not isolate themselves for 14 days should be reported to the appropriate authority	(86.4%)		
A5	Nigeria's level of preparedness in the fight against COVID-19 is commendable	(47.2%)		
Perception				
P1	SARS Cov-2 was not made in the laboratory	(46.8%)		
P2	The control of COVID-19 pandemic is a collective effort	(76.0%)		
P3	People are experiencing economic hardship due to the COVID-19 pandemic	(79.0%)		
P4	The use of medical mask can help protect you against COVID-19 infection	(78.0%)		
P5	COVID-19 truly exists and it is not a political propaganda	(80.4%)		
Practice				
Pr1	I avoid large gathering in recent days to reduce the risk of contracting COVID-19	(66.3%)		
Pr2	I wear surgical mask especially when going out during this trying time	(66.1%)		
Pr3	I frequently wash my hands with water and soap and avoid touching my face with unwashed hands these days	(88.2%)		
Pr4	I always ensure I maintain social distancing by keeping a distance of at least 1to 1.5 meters since this outbreak began	(66.1%)		
Pr5	I always ensure that I cover my mouth with my elbow or tissue paper when I want to cough or sneeze and put used tissue paper into dustbins these days	(32.4%)		

Key: F: False; IDK-I: Don't know; T: True.

The internal consistency of the questionnaire was measured using Cronbach's alpha coefficient before administration and was found to be 0.792.

### Statistical analysis

Correct answers for knowledge, attitudes, perceptions and practices were described using frequencies. Appropriate inferential statistics such as independent sample *t*-test, multivariate and univariate analysis of variance (MANOVA) were used to compare the scores of knowledge, attitudes, perception and practices with respect to the respondent's demographics. Pearson correlation analysis was used to identify correlation between the various KAPP scores. The significance level was set at p < 0.05. All data analyses were conducted using IBM SPSS version 22.0.

## Results

Seven hundred and thirteen respondents participated in the survey by accepting to fill in the questionnaire. Among the respondents, 385 (54.0%) are within the age range of 18–30 years, 273 (38.3%) are within 31–50 years of age and 55 (7.7%) are older than 50 years. The majority of the respondents (585) are residing in North West Nigeria, representing 82.0% of the participants. The remaining participants are residing in the North East (87) and North Central (41) regions representing 12.2 and 5.8%, respectively. Four hundred and nine (57.4%) of the respondents are married, while 279 (39.1%) and 25 (3.5%) are single and divorced, respectively. Out of the total participants, 483 (67.7%) were males while 230 (32.3%) were females. More so, 258 (36.2%) have bachelor's degree (HND/BA/BSc) and 206 (28.9%) are civil servants. The rest of the demographic characteristics of the respondents are presented in Table 2.

**Table 2.** Multivariate and univariate analysis of variance showing relationship between some demographic variables and participants' scores (mean ± SE) on knowledge, attitudes, perceptions and practices.

Variable	Level	Knowledge	Attitude	Perception	Practice
Age	18–30 years	10.8 ± 0.13	3.8 ± 0.06	3.3 ± 0.78	3.3 ± 0.75^a^
	31–50 years	12.4 ± 0.15	4.2 ± 0.07^a^	4.0 ± 0.09^a^	3.0 ± 0.09^b^
	Over 50 years	9.8 ± 0.33	4.3 ± 0.15^a^	4.1 ± 0.21^a^	3.5 ± 0.20^a,b^
Region	North West	11.3 ± 0.11^a^	3.9 ± 0.05^a^	3.4 ± 0.06	3.0 ± 0.06^a^
	North East	11.4 ± 0.28^a^	4.6 ± 0.12^b^	4.5 ± 0.16^a^	4.2 ± 0.15
	North Central	12.0 ± 0.40^a^	4.3 ± 0.17^a,b^	4.3 ± 0.23^a^	3.4 ± 0.22^a^
Marital status	Married	12.0 ± 0.12	4.4 ± 0.05	3.8 ± 0.08	3.3 ± 0.07^a,b^
	Single	10.7 ± 0.15	3.5 ± 0.06^a^	3.1 ± 0.09	3.1 ± 0.09^a,c^
	Divorced	8.8 ± 0.50	3.8 ± 0.21^a^	5.0 ± 0.30	3.2 ± 0.30^b,c^
Western education qualification	None	6.5 ± 0.60	3.1 ± 0.27^a^	5.0 ± 0.37^a^	2.2 ± 0.37^a^
	SS Cert.	10.9 ± 0.14^a^	3.7 ± 0.06^a^	3.1 ± 0.09	3.0 ± 0.08^a^
	Bachelor's Degree	11.4 ± 0.15^a^	4.3 ± 0.07^b^	3.6 ± 0.09	3.3 ± 0.09^b^
	Postgraduate	13.0 ± 0.21	4.4 ± 0.09^b^	4.5 ± 0.13^a^	3.6 ± 0.13^b^
Occupation	Civil Servant	12.6 ± 0.18	4.2 ± 0.08^a^	4.3 ± 0.11	3.3 ± 0.10^a^
	Business Owner	10.8 ± 0.20^a^	4.4 ± 0.09^a^	3.5 ± 0.12^a^	3.4 ± 0.11^a^
	Unemployed	10.9 ± 0.13^a^	3.7 ± 0.06	3.2 ± 0.08^a^	3.0 ± 0.08
Gender	Male	11.2 ± 0.13	4.0 ± 0.05^a^	3.8 ± 0.06	3.3 ± 0.06
	Female	11.8 ± 0.14	4.1 ± 0.08^a^	3.2 ± 0.12	3.0 ± 0.12

Means with the same superscript are not significantly different (p < 0.05).

SE: Standard error of mean; SS Cert.: Senior secondary certificate.

The ranges of the correct answers to questions on the knowledge, attitude, perception and practice in the questionnaire were from 51.8 to 95.1%, 47.2 to 92.6%, 46.8 to 80.4% and 32.4 to 88.2%, respectively (Table 1). The COVID-19 mean knowledge score significantly differed (p < 0.05) among age groups with 18–30 years, 31–50 years and 50 years and above having mean scores of 10.8 ± 0.13, 12.4 ± 0.15 and 9.8 ± 0.33, respectively (Table 2). With respect to region, the mean scores obtained for those living in North West, North East and North Central Nigeria were 11.3 ± 0.11, 11.4 ± 0.28 and 12.0 ± 0.40, respectively (Table 2) without any significant difference (p > 0.05). Marital status was found to play a significant role in the knowledge score of the respondents. Married respondents had a mean knowledge score of 12.00 ± 0.12 compared with the single or divorced respondents whose mean scores were 10.7 ± 0.15 and 8.8 ± 0.50, respectively, with a significant difference (p < 0.05). The education qualification of the respondents revealed that, the mean knowledge score of those with SSCE (senior secondary certificate)/primary education certificates (10.9 ± 0.14) did not differ significantly (p > 0.05) with those with bachelor's degree (11.4 ± 0.15) only, but a statistical difference (p < 0.05) does exist with the mean knowledge score of those with no Western education (6.5 ± 0.60) and those with postgraduate degrees (13.0 ± 0.21). The mean knowledge score of civil servants (12.6 ± 0.20) differ significantly (p < 0.05) with that of business owners (10.8 ± 0.20) and unemployed (10.9 ± 0.13). Student's *t*-test analysis indicated a significant difference (p < 0.05) between the male (11.2 ± 0.13) and female (11.8 ± 0.14) knowledge scores.

The majority of the respondents (87.2%) believed that social distancing can help stop the outbreak of COVID-19, while 92.6% believed that frequent hand washing with sanitizers or soap will break the transmission of the disease. Similarly, 87.2% answered that it is true most of the hospitals in Nigeria are not adequately equipped to handle patients with severe form of COVID-19 and 86.4% of the respondents agreed that people with recent history of overseas travel who did not isolate themselves should be reported to the appropriate authority. However, relatively few people (47.2%) agreed that Nigeria's level of preparedness in the fight against COVID-19 is commendable. On the attitude score, the mean score of those between the ages of 18 and 30 was found to be 3.8 ± 0.6 and differed significantly (p < 0.05) with the attitudes of respondents within the 31–50 (4.2 ± 0.07) and 50 and above (4.3 ± 0.15) age groups. However, no significant difference exists between the attitudes of respondents from North West (3.9 ± 0.05), North Central (4.3 ± 0.17) and North East (4.6 ± 0.12) except between North East and North West where a significant difference (p < 0.05) exists. The mean attitude scores of single (3.5 ± 0.06) and divorced (3.8 ± 0.21) respondents did not statistically differ (p > 0.05) but they all significantly differ (p < 0.05) with the mean attitude score of married respondents (4.4 ± 0.05). The mean attitude scores of the respondents without Western education (3.1 ± 0.27) and those with SSCE/primary education (3.7 ± 0.06) did not differ statistically (p > 0.05), however, they all differed significantly (p < 0.05) with the mean scores of those with bachelor's degree (4.3 ± 0.07) or postgraduate training (4.4 ± 0.09). With respect to occupation, the mean attitude scores of civil servants (4.2 ± 0.08) and business owners (4.4 ± 0.09) did not differ statistically (p > 0.05), but a significance difference (p < 0.05) exists between them and the mean score of unemployed (3.7 ± 0.06). Moreover, no statistical difference (p > 0.05) was observed between male (4.0 ± 0.05) and female (4.1 ± 0.08) mean attitude scores (Table 2).

On the perception of people on the origin of the SARS-CoV-2 virus, only 46.8% of the respondents answered that it is true the virus was not made in the laboratory, but 76.0% believe that the control of COVID-19 pandemic is a collective effort. Furthermore, 78.0% of the respondents answered affirmatively that people are experiencing economic hardship due to the COVID-19 pandemic and 78.0 and 80.4% of the respondents affirmed that using medical mask can help protect against the infection, and COVID-19 truly exists and it is not a political propaganda, respectively. The respondents' mean perception score across the demographics studied indicated that those in the 18–30 years age group (3.3 ± 0.78) significantly differed (p < 0.05) with those in the 31–50 years (4.0 ± 0.09) and 50 years and above (4.1 ± 0.21) age groups, while respondents residing in the North West (3.4 ± 0.06) had a significantly different (p < 0.05) mean score compared with those residing in the North East (4.5 ± 0.16) or North Central (4.3 ± 0.23). Furthermore, a significant statistical difference (p < 0.05) was found to exist between respondents based on their marital status, with the mean score of married, single and divorced respondents being 3.8 ± 0.08, 3.1 ± 0.09 and 5.0 ± 0.30, respectively. Those respondents with no Western education (5.0 ± 0.37) and postgraduate training (4.5 ± 0.13) did not significantly differ (p > 0.05) in their mean perception scores, however, differ significantly (p < 0.05) with the mean scores of those with SSCE/primary education (3.1 ± 0.09) or bachelor's degree (3.6 ± 0.09). The mean perception score of civil servants (4.3 ± 0.11) significantly differ (p < 0.05) with business owners (3.5 ± 0.12) and unemployed (3.2 ± 0.08) who in turn did not differ significantly (p > 0.05). Last, the mean perception score of male respondents (3.8 ± 0.06) differ significantly (p < 0.05) with female respondents (3.2 ± 0.12).

Among the participants, 66.3 and 66.1% affirmed that they avoided large gathering in recent days to reduce the risk of contracting COVID-19 and that they wear a surgical mask especially when going out, respectively. Similarly, 88.2 and 66.1% of the participants affirmed that they frequently washed their hands with water and soap and avoided touching their face with unwashed hands and that they always ensured that they maintained social distancing of at least 1.5 meters since the outbreak began. However, only few of the participants (32.4%) affirmed that they ensured that they covered their mouth with elbow or tissue paper when they wanted to cough or sneeze and properly disposed the used tissue paper. The mean practice score indicated that those respondents over 50 years (3.5 ± 0.20) did not differ significantly (p > 0.05) with those in the 18–30 years (3.3 ± 0.75) and 31–50 years (3.0 ± 0.09) age groups who in turn differ among themselves significantly (p < 0.05). The mean practice score of respondents residing in the North East (4.2 ± 0.15) differ significantly (p < 0.05) with the scores of those in the North West (3.0 ± 0.06) and North Central (3.4 ± 0.22). No significant difference (p > 0.05) was found between the mean practice scores of married (3.3 ± 0.07) compared with the single (3.1 ± 0.09) and divorced (3.2 ± 0.30) respondents or when single (3.1 ± 0.09) and divorced (3.2 ± 0.30) respondents were compared. Similarly, the mean practice score of the respondents without Western education (2.2 ± 0.37) compared with SSCE/primary certificate holders (3.0 ± 0.08) or those with bachelor's degree (3.3 ± 0.09) compared with postgraduate degree holders (3.6 ± 0.13), respectively, did not differ statistically (p > 0.05), however, significant difference (p < 0.05) exist when uneducated or SSCE/primary certificate holders were compared with bachelor's degree or postgraduate degree holders. Civil servants (3.3 ± 0.10) and business owners (3.4 ± 0.11) did not differ significantly (p > 0.05) in their mean practice score, but both differ significantly (p < 0.05) with the mean score of unemployed (3.0 ± 0.08). Last, significant difference was found in comparison between the male (3.3 ± 0.06) and female (3.0 ± 0.12) mean practice scores (Table 2).

According to the Pearson correlation conducted to determine the relationship between the knowledge, attitude, perception and practice scores of the respondents, knowledge was more strongly positively related to attitude (r[711] = 0.622; p < 0.001) than to perception (r[711] = 0.454; p < 0.001) or practice (r[711] = 0.282; p < 0.001). On the other hand, attitude was found to be strongly positively related to perception, (r[711] = 0.567; p < 0.001) than to practice (r[711] = 0.470; p < 0.001), while perception was found to be strongly positively correlated to practice (r[711] = 0.538; p < 0.001) (Table 3).

**Table 3.** Pearson correlation between knowledge, attitude, perception and practice scores.

		Knowledge	Attitude	Perception	Practice
Knowledge	r =	1	0.622	0.454	0.282
	p =		<0.001	<0.001	<0.001
Attitude	r =	0.622	1	0.567	0.470
	p =	<0.001		<0.001	<0.001
Perception	r =	0.454	0.567	1	0.538
	p =	<0.001	<0.001		<0.001
Practice	r =	0.282	0.470	0.538	1
	p =	<0.001	<0.001	<0.001	
	n	713	713	713	713

n: Sample size; p: Precision; r: Ratio.

## Discussion

The literature search indicated that this is the first study in Nigeria that determined the KAPP of people residing in the whole 19 states of Northern Nigeria toward the COVID-19 pandemic. The study revealed that the majority of the people who participated in the study were males (67.8%), within the age range of 18–30 years (54.0%), married (57.4%), held bachelor's degrees (36.2%), civil servants (28.90%) and were residing in the North West region (82.0%). These findings were closely related with the findings of [11] in Kano, north-western Nigeria, who reported higher percentage of male participants (55.4%) and low percentage of civil servants (20%). There was an overall correct rate of 95.1% on the knowledge segment of the questionnaire, indicating a high knowledge of COVID-19 among the participants. The presence of highly correct COVID-19 knowledge rates among northerners may be expected because of the possible impact of the worldwide publicity the pandemic attracted through different outlets such as local and international media (television stations, radio stations, newspapers etc.), social media, posters and public awareness campaigns embarked upon by the government, professional organizations and non-governmental organizations in both English and local languages (Hausa, Fulfulde, Kanuri and others). This is evident by the fact that significant difference was found between high knowledge score and age group, gender, level of education, marital status and occupation. In the age grouping, those within the 18–30 years and 31–50 years are considered young within the northern community and are therefore more likely to be using social media outlets such as Facebook, Twitter, Telegram and WhatsApp than those within the age group of 51 and above, who by nature of the northern community settings will consider such outlets as inappropriate for them to use. These social media platforms have overtaken regular media outlets as a means of information dissemination to the public, prompting international and local public health organizations such as WHO and NCDC to adopt them for passing COVID-19-related information. This finding agreed with the reports of Zhong and Reuben *et al.* [9,12] who related the significant association between a high COVID-19 knowledge score and the level of education of the respondents in their study. The finding that 47.2% of the respondents believed that the level of preparedness against COVID-19 pandemic exhibited by the Nigerian authority is commendable is indicative that the media campaigns and the resources set aside by the government for the fight against COVID-19 is appreciated only by few, therefore, more needs to be done. According to many of the participants (87.2%), Nigerian hospitals are ill equipped to handle patients with severe form of COVID-19 infection, a view that testifies to the weakness of the country's healthcare system as pointed out by Garba *et al.* [13], which can be easily overwhelmed if adequate control measures are not taken to stem the spread of the disease thereby leading to healthcare crisis. Even advanced economies like the USA, Italy, Spain and France had their healthcare systems stretched beyond their capacities during this pandemic. Age, place of residence, marital status, level of education and occupational status of the respondents were found to be positive predictors of good knowledge score at different levels. Those within the 31–50 years age group were found to have a higher mean knowledge score (12.4 ± 0.15) than either the 18–30 years age group (10.8 ± 0.13) or the 50 years and above (9.8 ± 0.33) age groups. This may be due to the fact that the 31–50 years age bracket within the context of Northern Nigeria is the age group that constitute the most active and economically prosperous members of the society and can therefore afford to buy smart phones, TVs and radio sets, access to internet and other things that will make access to COVID-19 prevention protocols easier. The majority of people within the 18–30 years age group use smart phones to access information, however, money to buy data for internet connectivity may not be readily available for this age group as they quite often depend on their parents or relatives to provide access for these and many more services. The 50 years and above age group often rely on TV stations and newspapers as the most trusted and reliable way to access information on COVID-19 as many of them consider smart phones and social media outlets as services for young people which cannot be trusted as a source of information due to the frequent spread of fake news within the social media circles. The finding of married respondents having significantly higher mean knowledge score (12.00 ± 0.12) than either single (10.7 ± 0.15) or divorced (8.8 ± 0.50) respondents agreed with the findings of Habib *et al.* [11] who found that marital status was associated with good knowledge. This is not surprising within the northern settings as married couples have a higher sense of responsibility and obligation to one another than singles who in most cases have a reckless and dare-devil disposition or divorced who are often withdrawn and reclusive. As expected, having Western education was found to be associated with good knowledge score compared with those who had no Western education background, a finding that agreed with previous reports [11,12]. This is because Western education gives people the required ability to read, write and speak in English, which is the official language in Nigeria and therefore, the language of communication and information dissemination, although local languages were used for COVID-19 awareness campaigns. This may also be the reason why civil servants had higher mean knowledge score (12.6 ± 0.20) as they are mostly educated and can easily understand the dangers associated with COVID-19 infection compared with business owners (10.8 ± 0.20) or unemployed (10.9 ± 0.13) who constitute the vast majority of those without formal education.

The majority of the participants (92.6%) have a positive attitude toward COVID-19, believing that frequent hand washing with sanitizers can help break the chain of transmission of the disease, and that anybody with recent overseas travel who did not isolate from the public should be reported to the authorities so that they can be forcefully quarantined. This finding is higher than the 79.5% reported by [12] in the North Central region or the 17.8% reported by [11]. These positive attitudes will no doubt help to stem the spread of the disease within communities. Even though few participants (47.2%) believed that the level of preparedness against COVID-19 pandemic exhibited by Nigerian authority is commendable, many (87.2%) are of the view that Nigerian hospitals are ill equipped to handle patients with severe forms of COVID-19 infection, reflecting the poor health infrastructure and the need for health-system strengthening in the country. The findings of positive attitude in this study indicated the level of awareness and knowledge the northern populace has on COVID-19 and its current control and preventive measures as advocated by WHO and NCDC. This is evident by the fact that 87.2 and 92.6% of the respondents have recognized the crucial role social distancing and frequent hand washing with hand sanitizer plays in the fight against COVID-19, respectively. A finding that reflected the report of [11] who found 66% of the respondents willing to practice social distancing, it also agreed with the findings of [12] who reported 92.7, 96.4 and 82.3% practicing social distancing, improve their personal hygiene and wear face masks, respectively. Furthermore, many (86.4%) of the respondents understood the importance of self-isolation following recent overseas travel to the extent that they believed such persons who did not isolate themselves after recent travel history should be reported to the constituted authority so that they can be forced into the mandatory 2 weeks isolation as enshrined in the NCDC travel guidelines. This is in agreement with the findings of [9] who found positive optimistic attitudes toward COVID-19 in their study, and with the findings of [12] where 92.7% of the respondents in that study agreed to adhere to the self-isolation protocol after overseas travel.

The perception of almost half of the respondents (46.8%) that COVID-19 was made in the laboratory indicates the level of damage propaganda and unverified news can have on the perception of people toward events as they unfold in the world. This was earlier reported by [11] who observed that 48% of the respondents in that study did not agree that COVID-19 had animal origin and 36% believed that the virus was man-made. This is not surprising because there are many rumors circulating through social media outlets in Nigeria and overseas that the virus responsible for COVID-19 pandemic was made in the laboratory, this rumor was further aggravated by many religious and ill-informed scholars who are misinforming the public that the virus is not a living thing but a mere combination of some chemicals that are formed in the laboratory. This resulted in the public having suspicion that this pandemic is nothing but American and Chinese creation designed to depopulate Africa or prevent them from practicing their religion, a reason that may be connected with the findings of [11] where about 50% of the respondents in their study insisted on attending the weekly congregational Friday prayers despite the pandemic, a view held by the vast majority of people in the Northern Nigeria who are mostly Muslims, based on personal observation of the authors. This finding indicates that government needs to do a lot in public enlightenment and must put in place strict laws that will deter people against the spread of false rumors and unverified information. Despite the above findings, it is a good thing that 76.0, 78.0 and 80.4% of the respondents believed that COVID-19 pandemic control is a collective effort, wearing face masks will protect against infection and the disease truly exists, respectively. This perception would enhance adherence to COVID-19 prevention protocol and avert or reduce community transmission of the disease [13].

The avoidance of large gathering and wearing of surgical mask, especially when going out were among the practices studied and 66.3 and 66.1% of the respondents said they engaged in such good practices, respectively. Similarly, 88.2% said they wash their hands frequently and with water and soap and 66.1% said they always ensure they maintain social distancing since the outbreak began. These may be a reflection of the good knowledge and attitude the respondents have on COVID-19, however, only few number of the participants (32.4%) adopt the habit of covering their mouth with their elbow or tissue paper when they want to cough or sneeze and properly dispose the used tissue paper, a practice that may jeopardize the success if any recorded in the control of the disease within the northern society. These unprotective coughing and sneezing practices may be attributed to the religious mindset of the northern populace where they link everything with divine origin [11]. A significant difference was found between age, area of residence, level of education and gender of the respondents with respect to their mean practice score which may be connected to the high knowledge of COVID-19 and good attitudes and perspectives these same categories of respondents have on COVID-19, since high knowledge and good attitude toward something will definitely influence one's practices for or against it as observed by [9]. However, Pearson correlation analysis conducted in this study to determine the relationship between knowledge, attitude, perception and practice found stronger positive (r = 0.622; p < 0.001) relationship between knowledge and attitude followed by perception (r = 0.454; p < 0.001) and lastly, practice (r = 0.282; p < 0.001), while at the same time, attitude was found to be strongly positively related to perception (r = 0.567; p < 0.002) than to practice (r = 0.470; p < 0.001), while perception was strongly related to practice (r = 0.538; p < 0.001). This means that the higher the COVID-19 knowledge of the respondents, the higher the positive attitude they will have toward it which in turn will highly influence their perception of the disease to be positive that will enhance their good practices on prevention and control measures or protocols.

The high KAPP observed may be overestimated since the participants in this study consisted largely of men, highly educated civil servants within 18–30 years age group and residing in the north-west region. Putting into consideration that level of education and occupational status are proportionately related to the socio-economic status, the findings in this study can be generalized only to the northerners of high socio-economic status, hence a limitation of this study. By virtue of the culture in the northern society, older people and those with low socio-economic status are more likely to have limited access to internet and online resources and information on COVID-19, therefore, they are more likely to have poor knowledge, attitudes, perspectives and practices toward the disease, although it may not always be the case as found in this study. These vulnerable groups should thus be the focus of government health education enlightenment programs for effective control and prevention of community based transmission of COVID-19. Another limitation of this study is that the study was conducted from March to April 2020, when very little was known regarding the COVID-19 pandemic and SARS-CoV-2 the causative agent but was not published until now when a lot is now known about the disease, although the information this study addressed is still not known in the study area.

## Conclusion

Our findings suggest that young, male, married northerners of high socio-economic status had better knowledge, attitudes, perspectives and appropriate practices toward COVID-19 pandemic outbreak control and prevention strategies in Northern Nigeria.

## Future perspective

There is the need to study the old, uneducated and unemployed Northern Nigerian population to determine their knowledge, attitude, perception and practice on the COVID-19 pandemic since these cohorts were poorly represented in this study. Furthermore, vaccine hesitancy is another area of possible exploration in the future.

**Summary points**

- Questionnaire instrument was adapted and modified.
- The questionnaire was validated by a panel of experts.
- The questionnaire was tested for internal consistency using Cronbach's alpha coefficient.
- The questionnaire was self administered both online and physically.
- Data generated from the responses were coded, entered in IBM SPSS and analyzed.
- Young, male, married educated and employed respondents constituted the majority of the participants.
- Positive correlation was found between knowledge and attitude, perception and practices.
- Married and educated participants displayed better knowledge, attitude, perception and practice scores.
- This study covered the entire 19 Northern Nigerian states.
